# Motorcycle-Related Fatalities: A Systematic Review and Structured Narrative Synthesis

**DOI:** 10.7759/cureus.112266

**Published:** 2026-07-08

**Authors:** Athina Tousia, Nikolaos Kalogrias, Konstantinos Katsos, Chara Spiliopoulou, Emmanouil I Sakelliadis

**Affiliations:** 1 Department of Forensic Medicine and Toxicology, National and Kapodistrian University of Athens, Athens, GRC; 2 Forensic Service of Athens, Ministry of Justice, Athens, GRC

**Keywords:** helmet use, injury epidemiology, motorcycle accidents, road safety, toxicology, two-wheeler fatalities

## Abstract

Two-wheelers, including motorcycles, account for a significant portion of road traffic fatalities. Although several measures, including alcohol and helmet legislation, have been established, mortality rates remain high, specifically in low- and middle-income countries. This systematic review aims to identify and synthesize factors associated with fatal outcomes in motorcycle crashes.

We conducted a systematic search across PubMed, Scopus, and Web of Science from 2014 to April 2026. The studies included were observational (cohort, case-control, and cross-sectional) and examined fatal motorcycle crashes. Data extraction centered on helmet use, substance use involvement, injury patterns, and demographic variables. Due to substantial heterogeneity, meta-analysis was not performed, and findings were synthesized using a structured narrative approach. Study quality was assessed using the Joanna Briggs Institute (JBI) methodology for systematic reviews.

Sixty-five (65) studies were included, including data from across the globe. Lack of helmet use appeared to be commonly identified among fatal cases. Alcohol, as well as other substance use, prior to riding were significantly correlated with mortality. Males aged 15-45 were identified as the most vulnerable demographic group, while elderly victims also demonstrated high mortality numbers, suggesting the presence of a two-spike distribution.

Motorcycle collision fatalities are a result of several, commonly modifiable, behavioral and environmental factors. The establishment of intervention strategies, including helmet laws, substance abuse prevention, infrastructure improvements, and age- and gender-targeted education, is crucial to reducing mortality.

## Introduction and background

According to the World Health Organization (WHO), Road Traffic Accidents (RTAs) are the 8th leading cause of death globally and the leading cause of death among people aged 5-29 years [1]. Over 1.35 million people succumb each year to road traffic crashes. Furthermore, over 28% of all worldwide RTA fatalities consist of motor two-wheel (MTW) collisions, while in low- and middle-income countries (Vietnam, Indonesia, and parts of India), this number shoots up to 50% [2]. Differences in socioeconomic status are important, since high-income countries tend to have fewer fatalities, probably due to stricter mandatory helmet laws, better road-maintenance infrastructure, and mandatory insurance and licensing [1]. Two-wheelers include bicycles (pedal- or electric-powered) and powered two-wheelers (PTWs), such as mopeds and motorcycles [3]. This review focuses specifically on motorcycle-related fatalities, while focusing specifically on fatal motorcyclist incidents and combining epidemiological, toxicological, and forensic data. Helmet use can reduce mortality by 42%, suggesting it is a major preventive factor in two-wheeled vehicle casualties [4]. In terms of cause of death, the most common fatal injuries include craniocerebral injuries, thoracic trauma, and femoral fractures [5]. Alcohol/substance use is another important factor in the causation as well as mortality. Throughout the literature, 20-40% of casualty cases tested positive for alcohol [6,7]. It is important to establish preventive measures such as random breath testing, universal helmet regulations, and a thorough review of licensing policies, as these appear to reduce motorcycle fatalities [8]. Despite extensive literature on motorcycle crashes and helmet effectiveness, fatal motorcycle injuries remain inconsistently synthesized across epidemiological, clinical, toxicological, and forensic domains. By integrating evidence from diverse study designs, this review aims to provide a more comprehensive understanding of the factors associated with fatal outcomes and to identify gaps relevant to prevention, forensic evaluation, and future research. Consequently, there is a need for a systematic synthesis focused specifically on motorcycle-related fatalities.

Protocol and registration

No protocol was registered with PROSPERO or any other prospective registry. This has been acknowledged as a limitation of the review. We conducted a systematic literature review using the Preferred Reporting Items for Systematic Reviews and Meta-Analyses (PRISMA) 2020 Guidelines [9].

Information sources

We searched databases including PubMed, Scopus, and Web of Science.

Search strategy

Search words included: “Motorcycle”, “Fatal”, “Mortality”, “Fatal Injury”, “Collision”. The literature search was initiated in January 2026 and completed on 10 April 2026. Studies published between 2014 and 2026 were considered eligible for inclusion in the review.

The PubMed search was performed using MeSH terms and Title/Abstract terms. The following search string was applied: (("Motorcycles"[Mesh] OR motorcycle[Title/Abstract] OR motorcyclist[Title/Abstract] OR motorbike*[Title/Abstract] OR "powered two-wheeler*"[Title/Abstract] OR "powered two wheeler*"[Title/Abstract]) AND (fatalit*[Title/Abstract] OR death*[Title/Abstract] OR mortality[Title/Abstract] OR "fatal injury"[Title/Abstract]) AND ("Accidents, Traffic"[Mesh] OR crash*[Title/Abstract] OR collision*[Title/Abstract] OR accident*[Title/Abstract] OR "road traffic"[Title/Abstract] OR "traffic injur*"[Title/Abstract])) AND ("2014/01/01"[Date - Publication] : "2026/04/30"[Date - Publication])**. For Scopus, the search was conducted in the Title, Abstract, and Keywords fields using the following query: TITLE-ABS-KEY ( motorcycle OR motorcyclist OR motorbike* OR moped* OR scooter* ) AND TITLE-ABS-KEY ( fatalit* OR death OR deaths OR mortality ) AND TITLE-ABS-KEY ( crash* OR collision* OR accident* OR injury OR injuries ) AND PUBYEAR > 2013 AND PUBYEAR < 2027**. These studies were retrieved from 2014 to 2026. Results were restricted to the subject area Medicine, document type Article, final publication stage, and English language.

The Web of Science search was performed using the Topic field. The following search string was applied: TS=(motorcycle OR motorcycles OR motorcyclist OR motorcyclists) AND TS=(fatality OR fatalities OR mortality) AND TS=(crash OR collision). In Web of Science Core Collection, results were restricted to articles published between 2014 and 2026 and refined by relevant Web of Science categories, including Transportation; Public, Environmental & Occupational Health; Surgery; Emergency Medicine; Medicine, General & Internal; Orthopedics; Critical Care Medicine; Tropical Medicine; Nursing; Health Care Sciences & Services; Pediatrics; Toxicology; Dentistry, Oral Surgery & Medicine; Infectious Diseases; Medical Informatics; Psychiatry; Substance Abuse; Anatomy & Morphology; Anesthesiology; Ethics; Clinical Neurology; Pathology; Medicine, Legal; Peripheral Vascular Disease; Health Policy & Services; Immunology; Social Sciences, Biomedical; Dermatology; Transplantation; Cardiac & Cardiovascular Systems; Respiratory System; Medical Ethics; Obstetrics & Gynecology; Oncology; Otorhinolaryngology; Primary Health Care; Radiology, Nuclear Medicine & Medical Imaging; Rehabilitation.

The database searches identified a total of 3,876 records before duplicate removal: 840 from PubMed, 945 from Scopus, and 2,091 from Web of Science Core Collection. Searches were limited to English-language articles published between 2014 and 2026. Search yields reflect the number of records retrieved on the original search date and may differ from later database reruns due to indexing updates.

Study selection

We removed duplicates and screened titles/abstracts using Rayyan (Rayyan Systems Inc., Cambridge, MA, US) [10]. 

The screening was performed by two reviewers. Titles and abstracts were independently screened by two reviewers. Full-text articles were then assessed for eligibility by the same reviewers. Disagreements were resolved through discussion. Inter-reviewer agreement was not formally quantified using kappa statistics. Subsequently, one reviewer reviewed the full texts of the eligible articles.

We used a pre-defined data extraction form that captured authorship, country, design of the study, number of cases (fatal), type of vehicle, types of injuries, toxicology results, use or not of helmets, and demographic characteristics. Finally, we grouped the findings by themes (demographic, injuries, and toxicology results). Missing or inconsistently reported variables were recorded as not reported.

Risk-of-bias assessment

This systematic review was conducted in accordance with the Joanna Briggs Institute (JBI) methodology for systematic reviews [11]. The appropriate JBI checklist was selected based on the study design, including tools for analytical cross-sectional, cohort, prevalence, case series, and quasi-experimental or time-trend studies, where applicable. Each item was rated as “Yes,” “No,” “Unclear,” or “Not applicable.” An overall appraisal was assigned to each study based on the proportion of fulfilled criteria. Overall, most included studies demonstrated moderate methodological quality, with common limitations including retrospective design, incomplete reporting of helmet use, and limited adjustment for confounding factors.

Large registry-based studies provided broader population-level data but often lacked detailed forensic information, whereas smaller forensic or hospital-based studies provided richer injury and toxicological detail but had limited generalizability. These limitations were considered when interpreting the findings. Study-level judgments were summarized in tabular form. Recurring methodological limitations were narratively synthesized and considered when interpreting the findings. Overall appraisal categories were not used as automatic exclusion criteria. Recurring methodological limitations included retrospective study design, incomplete reporting of key variables, inconsistent mortality definitions, and limited adjustment for potential confounding factors. These issues were considered when interpreting the findings.

Eligibility criteria

The population, exposure, comparator, and outcomes (PECO) framework was used for the inclusion criteria. The population of interest comprised the fatally injured motorcycle riders involved in road traffic injuries. The main exposures of interest were helmet non-use and alcohol or drug positivity, where reported. Comparators included helmeted versus non-helmeted fatal cases and alcohol/drug-positive versus alcohol/drug-negative fatal cases, where such comparisons were available. In studies without explicit comparator groups, findings were summarized descriptively. The primary outcome was motorcycle-related fatality/death. Age, sex, injury patterns, cause of death, toxicological findings, and other available forensic or crash-related information were extracted as descriptive variables rather than treated as exposures. For studies that included broader trauma cohorts or mixed road-user populations, only data specifically related to fatal motorcycle cases were extracted and considered for synthesis. Studies were excluded if fatal motorcycle data could not be separated from nonfatal cases or from other road-user groups. Mixed-population studies were included only when motorcycle-related fatality data were clearly extractable. Studies without separable motorcycle fatality data were excluded from the main synthesis or used only as background context where appropriate. The primary outcomes assessed were fatality and mortality. Studies were included if they were original studies involving fatal road traffic incidents with motorcycles and reported at least one relevant variable (e.g., age, sex, helmet use, toxicology, or injury patterns), and were published in English between 2014 and 2026. Exclusion criteria encompassed non-English-speaking literature, studies not focused on motorcycles, bicycle/e-scooter-only studies, nonfatal injury-only studies, Case reports, reviews, conference proceedings without full peer-reviewed publication, theses, news media reports, abstracts, citations, and articles that didn’t include full text.

Data extraction

Data were extracted using a standardized extraction form. Extracted variables included author, year, country, study design, sample size, number of fatalities, age, sex, helmet use, toxicological findings, injury patterns, and main findings. Data extraction was performed by one reviewer and checked for accuracy by a second reviewer. A qualitative narrative synthesis was conducted. Findings were grouped according to demographic characteristics, helmet use, toxicological findings, injury patterns, and crash-related variables.

Data synthesis and statistical analysis

Because of substantial methodological and clinical heterogeneity, formal meta-analysis was not performed. The included studies differed in design, data source, population, denominator definition, mortality definition, injury classification, toxicological testing protocols, helmet-use reporting, and adjustment for confounding. Standardized effect estimates such as adjusted odds ratios, relative risks, hazard ratios, and 95% confidence intervals were not consistently reported for the same exposure-outcome pairs. Therefore, pooled effect estimation was considered methodologically inappropriate and potentially misleading. Instead, a structured narrative synthesis was performed. Findings were organized according to predefined domains: demographic characteristics, helmet use, toxicological findings, injury patterns, crash circumstances, and mortality definitions. Where possible, descriptive quantitative summaries were provided as counts and proportions of studies reporting each variable. The findings should therefore be interpreted as recurring patterns among fatal motorcycle cases rather than pooled estimates of risk or causality.

## Review

Study selection

**Figure 1:**
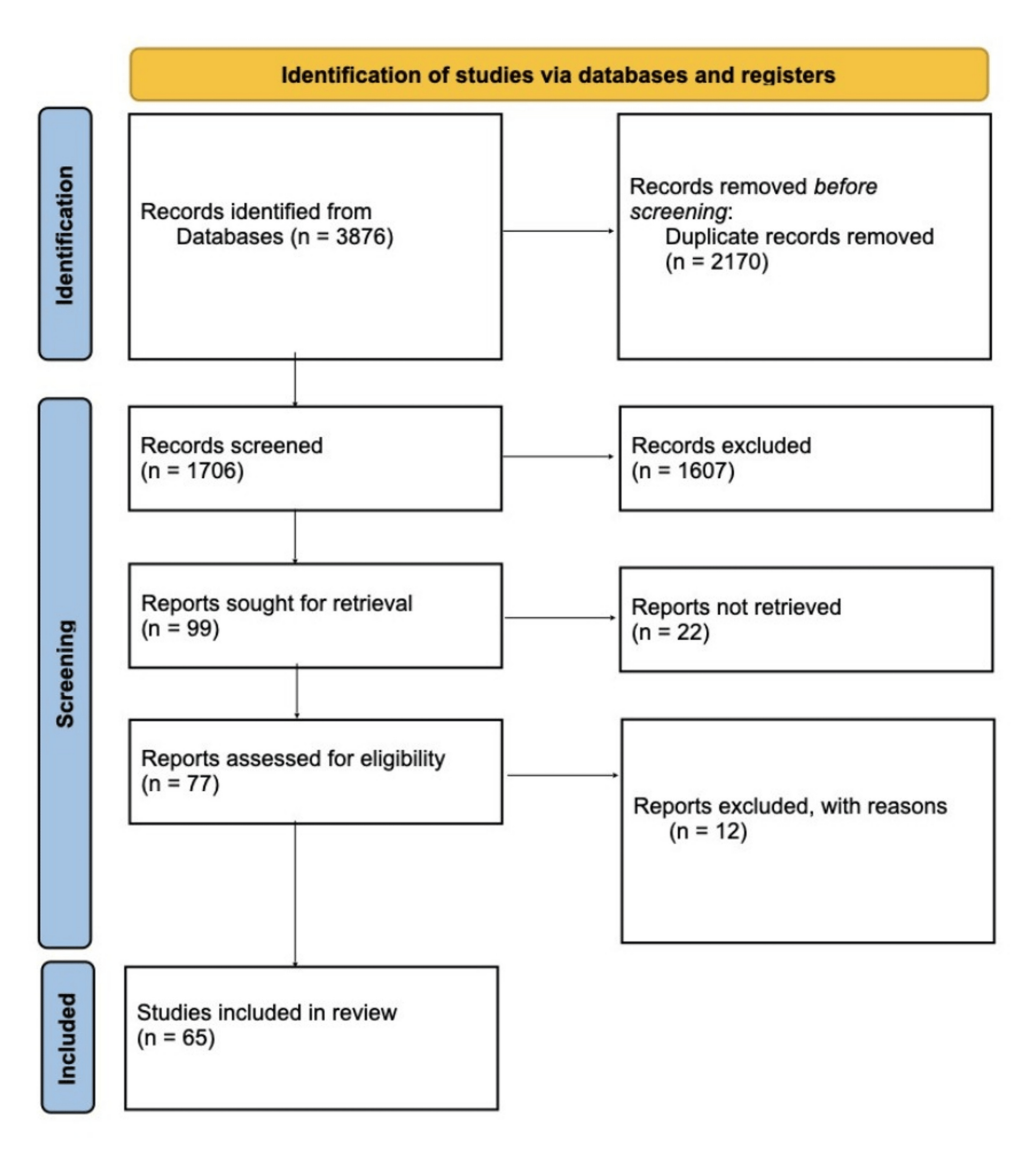
PRISMA flow chart PRISMA: Preferred Reporting Items for Systematic Reviews and Meta-Analyses

We conducted a systematic literature search across databases and identified numerous publications on fatal motorcycle incidents. After duplicate records were removed, the remaining articles underwent an initial screening of titles and abstracts. Studies unrelated to fatalities, those not involving motorcycles, or those lacking sufficient data were excluded at this stage. Subsequently, a full-text assessment of potentially eligible publications was performed. After removal of duplicates and title/abstract screening, 77 full-text articles were assessed for eligibility. After full-text assessment, 12 articles were excluded: Full-text articles excluded, with reasons (n = 12): Nonfatal outcomes only (n = 10), Review/editorial/commentary (n = 1), Full text unavailable (n = 1). Based on the pre-specified inclusion and exclusion criteria, 65 studies met the inclusion criteria and were included in the final analysis of this systematic review. The study selection process is presented in the PRISMA flowchart (Figure 1).

Characteristics of the included studies

The studies displayed considerable heterogeneity in design, sample size, and geographical distribution, with origins across Europe, Asia, the Americas, and Africa. The majority were retrospective epidemiological studies, analyses of national traffic accident databases, or hospital and forensic case series that focused on several factors contributing to mortality, including demographics, helmet use, substance use, and injury patterns. In several cases, substantial national traffic accident registries were used, including thousands of cases, while other studies were based on smaller case series from hospitals or forensic services. Most of the studies focused on motorcycle riders. The studies included in this systematic review are presented in Table 1. 

**Table 1:** Condensed summary of key characteristics and findings of the included studies aOR: adjusted odds ratio; BAC: blood alcohol concentration; GCS: Glasgow Coma Scale; ICU: intensive care unit; OR: odds ratio; RTA: road traffic accident; RTI: road traffic injury; TBI: traumatic brain injury Detailed study-level characteristics, including age, sex, helmet use, toxicological findings, and injury patterns, are provided in the Appendices.

Study	Country	Sample/fatal cases	Main focus	Condensed key finding
Takeda et al. (2020) [12]	Japan	Autopsy cases: 25 motorcyclists	Alcohol/toxicology	Alcohol associated with more severe injuries.
James et al. (2023) [13]	France	196 fatal motorbike cases	Alcohol/toxicology	Severe TBI was common; motorbike injuries were comparable in severity to bicycle/e-scooter injuries.
Gupta et al. (2014) [14]	India	2,718 victims	Helmet use/legislation	Helmet use was associated with lower fatal injury burden, while non-helmeted riders showed higher alcohol/drug involvement.
Rossheim et al. (2014) [15]	USA	9,861 motorcycle fatalities	Helmet use/protection	Drug use was associated with greater injury severity; helmet use remained protective.
Whyte et al. (2016) [16]	USA	47 fatal	Helmet use/protection	Full-face helmets showed greater protection for chin/jaw while the injury mechanisms differ by helmet type.
Lee et al. (2017) [17]	Taiwan	2,868 trauma patients (56 fatal)	Helmet use/protection	Helmet reduced severe head injuries by 34.5%, fatality by 1.3% and craniotomies by 7.8%.
Marya et al. (2017) [18]	India	Trauma patients (motorized two-wheelers) 4,297	Helmet legislation/helmet use	Legislation increased helmet use and significantly reduced incidence of head injury.
Rice et al. (2017) [19]	USA	7,189 riders	Helmet use/protection	Novelty helmets had 1.95× risk of death compared to full-face helmets.
Lee JM (2018) [20]	USA	National dataset (US)	Helmet legislation/helmet use	Universal helmet laws were associated with lower fatality, speeding, and risky driving.
Rajesh et al. (2019) [21]	India	303 RTA autopsies	Helmet use/protection	Higher fatality in unhelmeted, especially young males and crashes that took place at nightime.
Choi et al. (2020) [22]	South Korea	2,549 motorcycle crash patients	Helmet use/protection	Helmet reduced in-hospital mortality (aOR 0.39).
Notrica et al. (2020) [23]	USA	State-level data	Helmet legislation/helmet use	States with helmet laws had lower fatality rates.
Sarmiento et al. (2020) [24]	USA (Florida, Miami-Dade County)	227 fatal cases (2009–2014)	Helmet use/protection	Positive toxicology was linked to rider fault, single-vehicle crashes, and lower helmet use.
Hamnett et al. (2017) [25]	Hawaii, USA	Drivers involved in 102 fatal motorcycle crashes, 32 (27%) were motorcyclists	Alcohol/toxicology	Alcohol/drug positivity was frequent, often above legal BAC, with common combined substance use.
Ahmed et al. (2020) [26]	USA	67,183 motorcycle crash victims	Helmet use/protection	High BAC was associated with increased need for ICU, ventilation, longer hospital stay and no helmet use. High BAC is also a predictor of poor outcome.
Ramli et al. (2014) [27]	Malaysia	177 fatal cases	Alcohol/toxicology	Head trauma was the most common cause of death.
Paryavi et al. (2015) [28]	Iran	110 injured motorcyclists, 4% mortality	Injury patterns/trauma severity	Young males were most frequently affected; upper-limb fractures predominated.
Faduyile et al. (2017) [29]	Nigeria	156 fatal medico-legal autopsy cases	Helmet use/protection	Head injury dominant; higher fatality without helmet.
Hosseinpour et al. (2017) [30]	Iran	Motorcyclists injured or killed 2008–2015	Injury patterns/trauma severity	Injury frequency peaked in December and January.
Granieri et al. (2020) [31]	Italy	1,725 motorcycle trauma patients (mortality rate 4.9%)	Injury patterns/trauma severity	Head trauma had the highest fatality; chest and abdominal injuries increased risk.
Saman et al. (2021) [32]	Malaysia	126 autopsy cases	Alcohol/toxicology	Lower limb fractures most common; fatalities mainly from head injuries.
Canzi et al. (2022) [33]	Italy	6,069 motorcycle crash victims (2.4% mortality overall)	Injury patterns/trauma severity	Facial trauma strongly associated with mortality (OR 2.7); often co-occurs with TBI.
Slović et al. (2023) [34]	Serbia	525 trauma cases incl. 12 motorcycle cases	Injury patterns/trauma severity	Motorcycle crashes more likely to cause severe abdominal trauma and liver rupture.
Saunders et al. (2019) [35]	USA	71 fatal cases	Alcohol/toxicology	Most victims were male; TBI was the leading cause of immediately fatal crashes.
Lotfi et al. (2019) [36]	Iran	6,216	Epidemiology/mortality trends	Mortality higher in males, self-employed, low education; 54% died on scene.
Eraybar et al. (2019) [37]	Turkey	Not specified in extraction table	Injury patterns/trauma severity	Higher extremity injuries in motorcycles; 69.9% extremity trauma.
Vieira et al. (2019) [38]	Brazil	Not specified in extraction table	Epidemiology/mortality trends	278% increase in deaths; males 20–29 most affected.
Bakovic et al. (2019) [39]	Croatia	163 fatal cases	Alcohol/toxicology	Alcohol significantly associated with fatal crashes; responsible riders had higher BAC; weekend crashes more frequent.
Rappole et al. (2019) [40]	USA	2,852 crashes, 17% fatal (~485 deaths)	Helmet use/protection	Alcohol use and helmet non-use significantly increased risk of fatal injury.
de Souza et al. (2019) [41]	Brazil	1,458 fatal cases	Epidemiology/mortality trends	Pre-Law: mortality increased; Post-Law (Lei Seca): male mortality declined by 11.2%; clusters found in inner regions.
Roehler et al. (2015) [42]	Cambodia	174,103 crashes (2000–2006)	Helmet use/protection	alcohol and no-helmet use increase fatality.
Polanco et al. (2016) [43]	USA	144,151 fatalities	Epidemiology/mortality trends	Baby-boomers, at age 20-24, experienced the highest mortality rates from motorcycle crashes while continuing experiencing high mortality after age 40.
Umbare & Khetre (2017) [44]	India	95 autopsies	Injury patterns/trauma severity	Young adult males were the most affected group; none wore a helmet.
Bazargani et al. (2018) [45]	Iran	1,840 fatal cases	Injury patterns/trauma severity	Head trauma was the main cause of death.
Kuo et al. (2018) [46]	Taiwan	(168 fatal)	Helmet use/protection	Helmet use and female gender are key predictors of mortality in crash victims.
Halbersberg & Lerner (2019) [47]	USA	33 fatal cases	Alcohol/toxicology	Younger riders more likely to be involved in high-speed and alcohol-related crashes.
Lip et al. (2019) [48]	Brazil	1,653 (142 fatal)	Injury patterns/trauma severity	Head injuries most common.
Jomar et al. (2019) [49]	Brazil	Not exact, based on national registry trends	Alcohol/toxicology	Zero-tolerance law reduced motorcycle crash mortality among young riders.
Kim et al. (2019) [50]	South Korea	700 injured motorcyclists	Helmet use/protection	Non-helmeted riders had higher severity; head injury major cause of death.
Kukde et al. (2019) [51]	India	110 fatal	Helmet use/protection	Most deaths were due to head injury; low helmet use and alcohol were prominent risk factors.
Chang et al. (2020) [52]	Taiwan	266 fatal cases	Injury patterns/trauma severity	Motorcyclists had highest injury rate; male drivers had higher fatality rates.
Barzegar et al. (2020) [53]	Iran	28,356 motorcycle traffic fatalities (77.4% riders)	Injury patterns/trauma severity	Fatalities were predominantly male and young adults; head trauma was the leading cause of death.
Lu et al. (2020) [54]	USA	60 fatal	Alcohol/toxicology	Approximately 32% of deaths potentially preventable.
Sae-Tae et al. (2020) [55]	Thailand	1,654 fatal cases	Helmet use/protection	Among motorcycle users, male gender, older age, and not wearing a helmet increased mortality rates.
Genowska et al. (2021) [56]	Poland	24,910	Epidemiology/mortality trends	Higher mortality in low-resource areas and more out-of-hospital deaths. Poor healthcare access was a key factor.
Rahmanian et al. (2021) [57]	Iran	558 motorcycle cases, 18 fatal	Alcohol/toxicology	Head/neck, thoracic, abdominal, vertebral and extremity injuries, plus abnormal creatinine, predicted hospital mortality.
Pervez et al. (2022) [58]	Pakistan	354 fatal	Helmet use/protection	Fatal crashes were associated with helmet non-use, night riding, curves, speed violations, heavy motorcycles, and young male riders.
Lusetti et al. (2022) [59]	Italy	350 fatal (59.4% motorcycles)	Alcohol/toxicology	Older age and alcohol associated with higher fatality risk.
de Souza et al. (2022) [60]	Brazil	11,343 fatal	Epidemiology/mortality trends	67.52% of the cases were concentrated in the group of young adults aged 18–39 years.
Wang MH (2022) [61]	Taiwan	1,586 fatal cases of motorcycle accidents (28%)	Alcohol/toxicology	Male sex and BAC positivity were associated with increased fatality risk.
Yasin et al. (2022) [62]	United Arab Emirates	162 (fatal)	Injury patterns/trauma severity	Policy changes assisted in reducing fatalities.
Jafari-Khounigh et al. (2023) [63]	Iran	2,553 cases fatal cases	Injury patterns/trauma severity	Seasonal pattern of fatalities.
Leitão et al. (2023) [64]	Brazil	5,293 motorcyclist deaths	Epidemiology/mortality trends	High male mortality; hospital costs significant.
Champahom et al. (2023) [65]	Thailand	2018-2020 dataset	Epidemiology/mortality trends	Older age, male sex, night crashes increase risk.
Aşırdizer et al. (2024) [66]	Türkiye	7,199 motorcycle fatalities	Injury patterns/trauma severity	The highest risk of death was observed among individuals aged 65 and older.
Chiangkhong et al. (2024) [67]	Thailand	2,775	Helmet use/protection	Peak fatalities occur after school hours and during school semesters; boys 10–14 most at risk, especially without helmets and under alcohol influence.
Unkuri et al. (2024) [68]	Finland	147 fatalities (77 motorcycle)	Injury patterns/trauma severity	Head injury was the most common cause of death; most crashes involved collision with another vehicle.
Nugroho & Masrika (2025) [69]	Indonesia	324 motorcycle TBI cases; 30 deaths	Alcohol/toxicology	Low GCS and delayed hospital admission predicted mortality.
Gürbüz et al. (2025) [70]	Middle East	721 cases; mortality 4.2%	Injury patterns/trauma severity	Mortality associated with severe head, thoracic and abdominal trauma.
Abulkhair et al. (2025) [71]	Qatar	12,644 RTI hospitalizations; 53 motorcycle deaths	Injury patterns/trauma severity	Motorcycle-related injuries increased despite overall decline in RTI mortality.
Kruger et al. (2025) [72]	Brazil	17,726 crashes; 406 deaths (including motorcyclists)	Alcohol/toxicology	Mortality increased despite reduced traffic; alcohol and nighttime crashes important contributors.
Tollazzi et al. (2025) [73]	Slovenia	138 fatal road accidents	Motorcycle crash fatalities	Riders causing crashes were most often aged 25–34, 45–54, and 55–64 years.
Celiksoz et al. (2026) [74]	Türkiye	2,445 cases	Alcohol/toxicology	Young male riders predominated; extremity and head injuries common.
Tousia et al. (2026) [75]	Greece	94 cases	Injury patterns/trauma severity	Chest, abdominal, neck, and multiple-site injuries were associated with poorer survival.
Gyaase et al. (2026) [76]	Ghana	40,322 motorcycle crashes, of which 9,213 were fatal	Epidemiology/mortality trends	Risk was higher in male older riders.

More detailed study-level information, including extended data on sample characteristics, helmet use, toxicological findings, injury patterns, and main findings, is provided in the Appendices. 

Demographic characteristics

Gender was examined in 60 of 65 studies. According to the studies, victims of motorcycle fatalities were predominantly male. Age was examined in most studies (64 of 65). The age groups most frequently affected were young and middle-aged adults, although in some studies an upward trend in incidents was noted among older adults. This underlines the presence of a two-spike distribution. 

Helmet use

Helmet use was one of the most frequently examined factors in the included studies. Helmet use was examined in 39 of 65 studies. Most findings indicated that not wearing a helmet was frequently reported among fatal cases. They reveal a consistent association between non-helmet use and fatal head injuries from motorcycle, bicycle, and scooter accidents. It should be noted that novelty helmets appear to be associated with a significantly higher risk of mortality compared to full-face helmets, underscoring that novelty helmet use not only doesn’t offer protection but is also linked to the worst outcomes. Conversely, helmet use was associated with significantly lower mortality rates. Despite the well-established benefits of helmet use, several studies have reported relatively low compliance rates, particularly in regions with less strict helmet legislation or limited enforcement.

Injury patterns

Injury patterns were examined in 41 of 65 studies. In terms of injuries, head trauma was noted as the most common cause of death across motorcycle and bicycle fatalities. Head injuries were the most common type of injury associated with fatal RTAs. Thoracic injuries such as rib fractures, pulmonary contusions, and major vascular injuries were also very prevalent across many studies. In addition, abdominal injuries, including liver and spleen ruptures, or other intra-abdominal organs, were described in several studies. Polytrauma, usually including craniocerebral, thoracic, and abdominal injuries, was a very common injury pattern in fatal motorcycle accidents as well. Many studies reported that these injuries are the leading cause of death for motorcycle riders and passengers. In addition, extremity injuries were also frequently reported. These injuries were often associated with high-energy collisions and could contribute significantly to mortality.

Toxicological findings

Toxicological findings were examined in 30 of 65 studies. According to the majority of the studies, alcohol as well as substance use were commonly identified among fatal cases. Alcohol has been analyzed in most studies. On the contrary, substance use of other CNS-acting substances hasn’t been generally commented on in the literature, since they were studied rarely, with the most common substances being THC, benzodiazepines, and amphetamines, which may affect driving ability and reaction time. These findings are suggestive of the contributing role of alcohol and substance use in mortality.

Discussion

This systematic review identified recurrent patterns among motorcycle-related fatalities, including male predominance, frequent head and thoracic injuries, variable helmet use, and alcohol or substance involvement in a subset of cases. However, these findings should be interpreted cautiously because the included studies differed substantially in design, population, data source, mortality definition, and reporting completeness. The review, therefore, provides a structured narrative synthesis rather than pooled causal estimates. Correspondingly, differences between studies may reflect variations in socioeconomic context, road conditions, helmet-law enforcement, alcohol use, trauma-care access, reporting practices, and study design.

In terms of demographics regarding motorcycle fatalities, a male predominance trend was observed in accordance with most of the studies. According to both behavioral and epidemiological research, it has been proposed that male sex is connected to less-utilized safety precautions (such as wearing a helmet) and participating in dangerous behaviors like speeding and driving when under the influence of CNS-acting substances [52,77]. On the other hand, females showed lower rates of motorcycle-related fatalities across all age groups. Regarding the most affected age groups, young men, particularly those aged 18 to 40, appeared to be at greatest risk [40,41,76]. Again, a combination of behavioral and psychological variables (risk-taking, inexperience, distracted driving, not wearing protective gear, and the potential to drive while under the influence of alcohol) has been proposed to be associated with to increase the likelihood that adolescent motorcycle riders are involved in RTAs [78]. This point is not directly applicable to the present study, as youth-related risk-taking behavior was not specifically assessed in our review. However, as several studies also reported an increase in mortality among older riders, a double-peak distribution of risk was also identified. Older riders were not necessarily overrepresented in crash occurrence, but they appeared more vulnerable to fatal outcomes, potentially reflecting age-related frailty, reduced physiological reserve, and comorbidities [79-82]. Moreover, studies have suggested that more experienced drivers can at times overestimate their driving abilities, thereby contributing to higher fatality rates [73]. Again, these factors were not specifically assessed in the included studies and therefore fall outside the scope of the present review. The use of larger, more powerful motorbikes, overestimation of competence, delayed response time, and sensory deterioration are other variables that contribute to this double-pick distribution [83].

The use of protective helmets is recognized as one of the most effective preventive measures against fatal motorcycle incidents. In all studies analyzing helmet use, this was confirmed: Helmet non-use was more frequently reported among fatal cases. More specifically, helmet use is connected to a lower occurrence of craniocerebral injuries, which are considered the most common cause of death among victims of such vehicles [84]. In contrast, novelty helmet use appears to provide little or no meaningful protection and has been associated with worse outcomes [19]. Demographics are also important regarding helmet use, as younger men and drivers in developing countries have the lowest rates of compliance with the law. As stated before, alcohol use is found to be connected to the lack of helmet use, creating an interesting psychosocial interplay. It should be noted that in most studies, the exact helmet type is not specified (full-face vs. open-face). Nevertheless, helmet use is linked to lower mortality rates.

The present systematic review underlined the role of alcohol and substance consumption as an important possible contributing factor to fatal accidents [6,26,85]. Toxicology data of the studies used point to alcohol or drug consumption being linked to higher mortality rates [26,54,86,87]. Also, the use of alcohol has been connected to dangerous actions such as not wearing a helmet [15]. Polydrug use, a finding that complicates data extraction, may have additive or synergistic effects on driving performance, risk perception, reaction time, and injury risk [25]. Toxicology testing in fatal accidents is fundamental not only for epidemiological or forensic reasons but also for prevention tactics. Alcohol limit legislation, systematic alcohol testing, as well as testing for substances other than alcohol (benzodiazepines, cannabinoids, etc.), are essential for managing fatality [75]. Nonetheless, it should be noted that, in many cases, the time between the accident and the sampling was not included, which is crucial information for interpreting the data. To summarize, alcohol and substance use appear to be two of the most important modifiable and preventable factors, suggesting that the integration of toxicology testing in all the prevention stages is crucial for mortality mitigation.

In terms of injury patterns, polytrauma appears to be the most common injury pattern in fatal two-wheel accidents and usually includes craniocerebral, thoracic, and abdominal injuries [88]. The results from this review are directly connected to mechanisms of high-energy collisions, speed, and helmet use, as well as to demographic characteristics of the victims. Craniocerebral injuries are suggested to be the most common fatal type of injury, especially in victims without protective gear or helmets [29]. According to most studies, even in polytrauma cases, death was attributed to craniocerebral injury. Helmet use is linked to a reduction in both mortality rates and the severity of injuries [22]. Another very common site of fatal injuries is the chest, involving lung injuries, hemothorax, and pleural fractures, especially in accidents involving high speed and frontal collisions [32,89]. Abdominal injuries, although less common, are extremely dangerous, especially in cases of visceral rupture or hemorrhage due to vascular injury, since in several cases they are not diagnosed before death [31,34]. Furthermore, extremity injuries also appear to be very common [74], especially involving the lower extremities, and can, although they are rarely directly fatal, lead to hemorrhagic shock and worsen overall trauma severity. The existence of such injuries is a significant indicator of the severity of the injury mechanism.

Geographically, studies show variation in the most common type of injury and in helmet use. Beyond doubt, systematic documentation and analysis of injury patterns are essential not just for understanding the mechanisms but also for establishing preventive and diagnostic tools.

Limitations

This review has several limitations. First, the included studies were heterogeneous in design, population, geographic setting, data source, and mortality definition. Second, key variables such as helmet use, toxicological findings, crash circumstances, and injury patterns were inconsistently reported. Third, standardized effect estimates were not consistently available, preventing reliable meta-analysis. Fourth, most included studies were retrospective and observational, limiting causal inference. Fifth, the restriction to English-language publications may have introduced language bias. Sixth, no protocol was prospectively registered. Also, a formal Grading of Recommendations Assessment, Development and Evaluation (GRADE) [90] assessment was not performed because of the substantial heterogeneity in study designs, outcomes, mortality definitions, and the absence of consistently reported comparable effect estimates. Therefore, the certainty of evidence could not be formally graded, and the findings should be interpreted cautiously. Several potentially relevant variables, including fatality definition, prehospital versus in-hospital death, effect sizes, confidence intervals, adjustment variables, urban/rural setting, and speed-related factors, were not consistently reported across the included studies. As a result, these variables could not be uniformly extracted or synthesized across all studies. This inconsistent reporting represents an important limitation of the available evidence and reduces comparability between studies. A further limitation is that not all included studies were restricted to fatal cases. Some trauma registry and hospital-based studies included mixed fatal and non-fatal cohorts, although they were retained only when motorcycle-specific mortality data were extractable. Finally, because formal meta-analysis was not performed, no pooled effect estimates, heterogeneity statistics, sensitivity analyses, or publication bias assessments were calculated. The findings should therefore be interpreted as a structured synthesis of recurring patterns rather than pooled estimates of risk.

## Conclusions

This systematic review highlights the global and persistent burden of motorcycle-related fatalities. In conclusion, motorcycle collisions appear to mainly involve young men and are often associated with non-use of helmets as well as use of alcohol or drugs. Head injuries consistently emerge as one of the most common and significant findings in such cases, while injuries to the chest and abdomen, as well as polytrauma, also appear to be substantially linked to mortality. However, heterogeneity across studies and incomplete recording of key variables, such as toxicological findings, helmet use, and precise description of injuries, make it difficult to directly compare results. Due to substantial heterogeneity across the included studies, these findings should be interpreted as recurring patterns among fatal motorcycle cases rather than as pooled estimates of risk or causality. Therefore, more standardized studies and more comprehensive data collection are needed to enhance understanding of factors associated with fatal outcomes and support more effective prevention strategies.

## Appendices

**Table 2:** Papers included in the systematic review aOR: adjusted odds ratio; BAC: blood alcohol concentration; COD: cause of death; CNS: central nervous system; GCS: Glasgow Coma Scale; ICU: intensive care unit; IQR: interquartile range; NR: not reported; OR: odds ratio; RTC: road traffic collision; RTA: road traffic accident; RTI: road traffic injury; SD: standard deviation; TBI: traumatic brain injury; THC: delta-9-tetrahydrocannabinol This table demonstrates substantial heterogeneity in study design, sample size, data source, mortality definitions, and reporting of helmet use, toxicology, crash circumstances, and injury patterns.

Title	Authors	Year	Country	Sample	Age	Sex	Helmet use	Toxicology	Injuries	Main findings
Differences in severity of injuries between motorcyclist and bicyclist fatalities in single vehicle collisions [12]	Takeda et al.	2020	Japan	Forensic autopsy cases of 25 motorcyclists	Mean 62 yrs	Not reported	None wore helmet	Alcohol present in 52%	Severe neck and upper extremity trauma	Alcohol associated with more severe injuries
Comparison of Injuries Associated With Electric Scooters, Motorbikes, and Bicycles in France [13]	James et al.	2023	France	196 fatal motorbike cases	Median 33 years (IQR 25–46)	83.7% male	22.5% wore helmets	36.7% BAC above legal threshold	TBI most common cause of death	25.9% had severe TBI; e-scooter RTC mortality was 9.2%; injuries as severe as those from bicycles and motorbikes
Motorised Two-Wheeler Crash and Helmets [14]	Gupta et al.	2014	India	2,718 victims	Adults	90.51% male	1,323 helmeted / 1,395 non-helmeted	Alcohol/drug use 8.75% in non-helmeted vs. 5.51% in helmeted	Head injuries	No mortality for any helmeted female driver was reported for more than two consecutive years since 2011
Associations between drug use and motorcycle crash injury severity [15]	Rossheim et al.	2014	USA	9,861 motorcycle fatalities	Mean age about 40–42 years	96.4% male	63.2% helmeted	38.1% alcohol-positive, 10.3% marijuana-positive, 19.5% other-drug-positive	NR	Drug use was associated with higher injury severity while helmet use remained protective
Mechanisms of Head and Neck Injuries in Motorcycle Crashes [16]	Whyte et al.	2016	USA	47 fatal	Age range of 17 to 43 years	46 males	All helmeted	NR	Head/neck injuries	Full-face helmets showed greater protection for chin/jaw while the injury mechanisms differ by helmet type
How motorcycle helmets affect trauma mortality [17]	Lee et al.	2017	Taiwan	2,868 trauma patients (56 fatal)	39.6 ± 18.2 years in helmeted vs. 35.9 ± 22.4 in non-helmeted	Slightly higher helmet use in females than males	2,714 helmeted/154 non-helmeted	NR	Head injuries	Helmet reduced severe head injuries by 34.5%, fatality by 1.3% and craniotomies by 7.8%
Impact of compulsory helmet legislation on mortality rate [18]	Marya et al.	2017	India	Trauma patients (motorized two-wheelers) 4,297	Most victims 20–40 years	NR	NR	Helmet-law impact study. It does not report case-level helmet-use percentages	Head trauma	Legislation increased helmet use and significantly reduced incidence of head injury
Novelty helmet use and motorcycle rider fatality [19]	Rice et al.	2017	USA	7,189 riders	18–86 years, mean 38	94.9% male	Yes (Novelty vs. Full-face)	BAC ≥0.08 g/dL in 2.3%	Head 15.1%, neck 8.9%, torso 26.9%, extremities 66.0%	Novelty helmets had 1.95× risk of death compared to full-face helmets
Mandatory helmet legislation as tool for reducing motorcycle Fatalities [20]	Lee JM	2018	USA	National dataset (US)	39.6 ± 18.2 in helmeted vs. 35.9 ± 22.4 in non-helmeted	Slightly higher helmet use in females than males	2,714 helmeted/154 non-helmeted	NR	Non-helmeted riders had more head/neck and facial injuries; helmeted riders had more extremity injuries	Universal helmet laws reduce fatality by 20.5%, risky driving and speed also reduced
Motorcycle helmet use and its correlates in fatal crashes [21]	Rajesh et al.	2019	India	303 RTA autopsies	Unhelmeted fatalities mainly 20–29 years	83.02% male among unhelmeted fatalities	55.5% unhelmeted	NR	Head injury	Higher fatality in unhelmeted, especially young males and crashes that took place at nightime
Can helmet decrease mortality of crashed motorcyclists? [22]	Choi et al.	2020	South Korea	2,549 motorcycle crash patients	Mean 39.2 years non-helmeted vs. 41.9 years helmeted	Male predominance	Helmet use associated with lower mortality	Alcohol recorded; higher in non-helmeted group	Head injury	Helmet reduced in-hospital mortality (aOR 0.39)
Impact of helmet laws on motorcycle crash mortality rates [23]	Notrica et al.	2020	USA	State-level data	16–20, 21–55, 56–65, >65 years	Male sex associated with higher mortality	Universal helmet laws associated with markedly lower mortality	Alcohol involvement increased mortality	NR	States with helmet laws had lower fatality rates
Alcohol/Illicit Substance Use in Fatal Motorcycle Crashes [24]	Sarmiento et al.	2020	USA (Florida, Miami-Dade County)	227 fatal motorcycle crash victims (2009–2014)	Mean age 34.6 y (±13.5 y)	Male (n = 217, 95.6%)	Yes, significantly less in positive tox group (44% vs. 64%)	44.5% positive for alcohol/illicit substances	Most severe in head/neck (58.2%), chest (35%)	Positive tox group: higher fault rate (77% vs. 50%), more single-vehicle crashes (47% vs. 21%), less helmet use (44% vs. 64%)
Toxicological findings in drivers of fatal motorcycle crashes [25]	Hamnett et al.	2017	Hawaii, USA	Drivers involved in 102 fatal motorcycle crashes, 32 (27%) were motorcyclists	Mean age for both genders was 41.5.	88% male	Helmet use noted; no detailed subgroup analysis	53% positive for ethanol, 40% for drugs; most frequent: THC, benzodiazepines, amphetamines	NR	Alcohol and drug use highly prevalent; 63% of positive cases were above legal BAC. Combination of alcohol and drugs common in fatal crashes
Elevated blood alcohol impacts hospital outcomes in motorcycle injuries [26]	Ahmed et al.	2020	USA	67,183 Motorcycle crash victims presenting to hospital	Median 42–43 years	Predominantly male	BAC-positive riders were much less likely to be helmeted	BAC-positive above legal limit in 21.14% of tested riders	Polytrauma with focus on TBI and ICU needs	High BAC was associated with increased need for ICU, ventilation, longer hospital stay and no helmet use. High BAC IS Also a Predictor of poor outcome
Fatal injuries among motorcyclists in Klang Valley [27]	Ramli et al.	2014	Malaysia	177 fatal motorcycle crash victims	Fatal cases mainly 16–25 years	93.2% male	Mainly half-head/open-face helmets; only 2.8% explicitly documented as unhelmeted, but many unknowns	23.2% alcohol/illicit-drug positive; strongest predictor of death	Head (60.4%), chest (19.4%), neck (3.6%)	Head trauma was the most common cause of death
Upper extremity injuries in motorcyclists [28]	Paryavi et al.	2015	Iran	110 injured motorcyclists, 4% mortality	Median 39 years	88% male	70% helmeted	NR	Upper limb fractures (especially radius, ulna, humerus)	Young males most affected
Pattern of Injuries in Fatal Motorcycle Accidents [29]	Faduyile et al.	2017	Nigeria	156 fatal motorcyclist cases in medico-legal autopsies	Mostly 21–30 years	Predominantly male	NR	NR	Head (78%), thorax (30%), abdomen (18%), spine (10%)	Head injury dominant; higher fatality without helmet
Trend and Seasonal Patterns of Injuries and Mortality Due to Motorcyclists Traffic Accidents [30]	Hosseinpour et al.	2017	Iran	Motorcyclists injured or killed 2008–2015	Mean ~26.4 years; peak 20–29 years	89.3% male	NR	NR	Overall injuries classified by season and month	Injury frequency peaked in Dec and Jan
Motorcycle-related trauma: effects of anatomic injury patterns [31]	Granieri et al.	2020	Italy	1,725 motorcycle crash trauma patients (mortality rate 4.9%)	≤17, 18–54, ≥55 years; older riders had worse survival	The cohort was 90% male	NR	NR	Head, thorax, abdominal, extremities	Head trauma had highest fatality; chest and abdominal injuries increased risk
The Pattern of Injuries Among Motorcyclists [32]	Saman et al.	2021	Malaysia	126 cases-autopsy-based study	Young men most affected age group	Ratio of male to female of 19:1.	No difference between helmeted rider and nonhelmeted rider in skull fracture and intracranial hemorrhage	31% low prevalence of alcohol or illicit substance use	Lower limb (57.6%), head (27.1%), upper limb (10%)	Lower limb fractures most common; fatalities mainly from head injuries
The burden of facial trauma on mortality in motorcycle crashes [33]	Canzi et al.	2022	Italy	6,069 motorcycle crash victims (2.4% mortality overall)	35.4 ± 13.4 years	89.9% male	NR	NR	Limbs and head most commonly involved	Facial trauma strongly associated with mortality (OR 2.7); often co-occurs with TBI
Abdominal injuries in road traffic trauma [34]	Slović et al.	2023	Serbia	525 trauma cases incl. 12 motorcycle cases	16–92 years	Predominantly male	NR	NR	Liver, spleen, intestinal	Motorcycle crashes more likely to cause severe abdominal trauma and liver rupture
The danger zone: Injuries and conditions associated with immediately fatal motorcycle crashes in the state of Michigan [35]	Saunders et al.	2019	USA	71 fatal cases	Mean 41.1 years	84.5% male	57.7% helmeted before crash	25.4% alcohol, 4.2% illicit drugs	TBI and rib fractures were most common; 42.3% COD was TBI	Mostly male, mean age 41,1 years
Analysis and identification of the hidden relationships between effective factors in the mortality rate caused by road accidents [36]	Lotfi et al.	2019	Iran	6,216	Mainly 19–40 years	79% male	NR	NR	NR	Mortality higher in males, self-employed, low education; 54% died on scene
Comparison of Fatal Injuries Resulting from Tractor and High Speed Motorcycle Accidents in Turkey [37]	Eraybar et al.	2019	Turkey		Mean 31.3 years (motorcycle group)	90.6% male (motorcycle group)	NR	NR	Rates of head trauma and maxillofacial injury were similar between groups	Higher extremity injuries in motorcycles; 69.9% extremity trauma
Motorcycle accidents in Belo Horizonte and its Metropolitan Region: linear mortality trend from 2000 to 2012 [38]	Vieira et al.	2019	Brazil		Mainly 20–29 years	88.8%–96.5% male	NR	NR	NR	278% increase in deaths; males 20–29 most affected
Fatal motorcycle crashes in wide urban area of Zagreb, Croatia—A 10-year review [39]	Bakovic et al.	2019	Croatia	163 fatal cases	Mostly 20–39 years (64.2%)	95.7% male	NR	BAC > 0.5 g/kg in 53.8%; drugs in 10.4%	Multiple injuries (55.8%), isolated head trauma (23.3%).	Alcohol significantly associated with fatal crashes; responsible riders had higher BAC; weekend crashes more frequent
Factors associated with motorcycle traffic crash fatalities among active duty U.S. Army personnel [40]	Rappole et al.	2019	USA	2,852 crashes, 17% fatal (~485 deaths)	60% aged 20–29	97% male	95% wore helmets; not wearing helmet increased fatality risk	Alcohol use raised odds of death by 3.1×	79% of fatal injuries were sustained to the head and neck. Most injuries were fractures (60% fatal, 54% nonfatal)	Alcohol use and helmet non-use significantly increased risk of fatal injury
Mortality in motorcycle accidents in Alagoas (2001–2015) [41]	de Souza et al.	2019	Brazil	1,458 fatal cases	39% aged 20–29	91.3% male	NR	NR	NR	Pre-Law: mortality increased; Post-Law (Lei Seca): male mortality declined by 11.2%; clusters found in inner regions
Fatal motorcycle crashes: a growing public health problem in Cambodia [42]	Roehler et al.	2015	Cambodia	174,103 crashes (2000–2006)	Median age 34; increase in riders >40 years	Predominantly male	Lower in fatal crashes; helmeted riders had lower mortality	Alcohol in 1/5 of cases; drug presence not detailed	76% of fatalities sustained head injuries	alcohol and no-helmet use increase fatality
Mortality from motorcycle crashes: the baby-boomer cohort effect [43]	Polanco et al.	2016	USA	144,151 fatalities	54.5% were baby boomers	91.4% male	NR	NR	NR	Baby-boomers, at age 20-24, experienced the highest mortality rates from motorcycle crashes while continuing experiencing high mortality after age 40
Socio-Demographic profile of head injury victims [44]	Umbare & Khetre	2017	India	95 autopsies	Commonest age group affected was 21-30 years (36.8%)	Male to female ratio was 5.8:1	No one wore a helmet	NR	Head injuries	Ages 21-40
Epidemiology of Traffic Fatalities among Motorcycle Users in East Azerbaijan, Iran [45]	Bazargani et al.	2018	Iran	1,840 fatal cases	Mean ~32.3 years	90.2% male	35% helmeted in Iran and 10% helmeted in Pakistan	NR	Head and face (87.5%) followed by chest and abdomen (23.9%).	Head trauma main cause of death
Derivation and validation of different classification models to predict mortality in motorcycle crashes [46]	Kuo et al.	2018	Taiwan	(168 fatal)	Mean age ~39	30.4% male, 69.6% female	51.2% wore helmets	BAC was similar between survivors and non-survivors, with no statistically significant difference	Abdomen injuries in 82.1% of fatalities	Helmet use and female gender are key predictors of mortality in crash victims
Young driver fatal motorcycle accident [47]	Halbersberg et al.	2019	USA	33 fatal cases	Young drivers <25	All male	NR	Implied alcohol use effect	NR	Younger riders more likely to be involved in high-speed and alcohol-related crashes
Clinical characteristics of 1653 injured motorcycle drivers [48]	Lip et al.	2019	Brazil	1,653 (142 fatal)	Mostly 20–39	93% male	90% wore helmets	NR	Head injuries in 55.64% of fatalities	Head injuries most common
Effect of the zero-tolerance drinking and driving law on fatal motorcycle crashes [49]	Jomar et al.	2019	Brazil	Not exact, based on national registry trends	Young adults most affected	Predominantly male	NR	Law aimed at alcohol	NR	Zero-tolerance law reduced motorcycle crash mortality among young riders
Comparison of epidemiology and injury severity in motorcycle crashes [50]	Kim et al.	2019	South Korea	700 injured motorcyclists	Mostly 20–49	Over 90% male	68% wore helmets	Not reported	Skull fractures and intracranial injuries were the most common injuries among fatalities	Non-helmeted riders had higher severity; head injury major cause of death
Study of Motorcycle Fatalities In Mumbai [51]	Kukde et al.	2019	India	110 fatal	21–40 most frequent	95% male	72.6% used helmets	14.2% positive for alcohol	NR	Majority deaths from head injuries; low helmet use and alcohol prominent risk factors
Risk of Injury and Mortality among Driver Victims Involved in Single-Vehicle Crashes in Taiwan [52]	Chang et al.	2020	Taiwan	266 fatal motorcycle cases	18-29 most affected age group	Gender-specific analysis (majority male)	NR	NR	NR	Motorcyclists had highest injury rate; male drivers had higher fatality rates
Epidemiologic study of traffic crash mortality among motorcycle users in Iran (2011-2017) [53]	Barzegar et al.	2020	Iran	28,356 motorcycle traffic fatalities (77.4% riders)	All age groups included	Both	Helmet use 37.4%	NR	Head trauma main cause of death	95.3% were male and 4.7% were female, age group of 18-24 years (29.1%). Head trauma major cause of death (59.0%)
Redefining Preventable Death Potentially Survivable Motorcycle Scene Fatalities as a New Frontier [54]	Lu et al.	2020	USA	60 fatal	The mean (SD) age was 34.3 (±14.5) years	95% male	55% helmet	Alcohol/drugs in 50% of preventable deaths	The main causes of death were traumatic brain injury (TBI) (22/60) and exsanguination (23/60)	Approximately 32% of deaths potentially preventable
Determinants of severe injury and mortality from road traffic accidents among motorcycle and car users in Southern Thailand [55]	Sae-Tae et al.	2020	Thailand	1,654 fatal motorcycle cases	Age group less than 25 years (41.5%) the majority of motorcycle cases	88.8% male	Without a helmet (81.9%)	NR	NR	Among motorcycle users, male gender, older age, and not wearing a helmet increased mortality rates
Health Care Resources and 24,910 Deaths Due to Traffic Accidents: An Ecological Mortality Study in Poland [56]	Genowska et al.	2021	Poland	24,910	Age groups 25–44 (29.6%) and 45–64 (31.3%)	77.7% male	NR	NR	NR	Higher mortality in low-resource areas and more out-of-hospital deaths. Poor healthcare access was a key factor
Risk Factors of Mortality following Road Accident in Southern Iran [57]	Rahmanian et al.	2021	Iran	558 motorcycle cases, 18 fatal	Mean 27 years	No significant relationship between gender and hospital mortality	Not directly reported	44,7% alcohol use, 14,9% drugs	Head and neck injury the most common	Head and neck injury, thoracic injury, abdominal injury, vertebral injury, extremity injury and abnormal creatinine were independently associated with hospital mortality in injured patients
What Factors Would Make Single-Vehicle Motorcycle Crashes Fatal? [58]	Pervez et al.	2022	Pakistan	354 fatal	Most victims aged 20–29	97.2% male	Helmet use reduced fatality risk	NR	NR	Fatal crashes associated with no helmet use, night crashes, curves, speed violations, heavy motorcycles, and male riders aged 20–29
Over 30-year retrospective analyses of moped-motorcycle fatal road accidents in the northern area of the Italian region of Emilia Romagna and review of the literature [59]	Lusetti et al.	2022	Italy	350 fatal (59.4% motorcycles)	Average of 35.4 years old	89.4% male	58.0% wore a helmet	Alcohol in 25,5%	Extremities (90.50%), head (87.40%) and thorax (85.70%)	Older age and alcohol associated with higher fatality risk
Trend of traffic accident mortality rate among motorcyclists in the state of São Paulo, Brazil [60]	de Souza et al.	2022	Brazil	11,343 fatal	18 - 24 years (27.9%)	Men 88.1%	NR	NR	NR	67.52% of the cases were concentrated in the group of young adults aged 18–39 years
Investigating the Difference in Factors Contributing to the Likelihood of Motorcyclist Fatalities in Single Motorcycle and Multiple Vehicle Crashes [61]	Wang MH	2022	Taiwan	1,586 fatal cases of motorcycle accidents (28%)	41.8% 25-54 years old	62.7% of drivers were male	Fatality risk increased with helmet nonuse	Fatality risk increased with positive BAC	NR	Male motorcyclists, and riding with BAC values of less than the legally limited value increase motorcyclist fatality
Reduction of motorcycle‑related deaths over 15 years in a developing country [62]	Yasin et al.	2022	United Arab Emirates	162 (fatal)	Ages 4–64	97.06% men	NR	NR	Increased severity of head injuries, upper extremities injuries most common	Policy changes assisted in reducing fatalities
The Pattern of Motorcyclists' Death Due to Accidents and a Three-year Forecast in East Azerbaijan Province, Iran [63]	Jafari-Khounigh et al.	2023	Iran	2,553 cases fatal motorcycle cases	Average age 33.38 years	Mainly male	NR	NR	Head injury was the leading cause of death (70.8%)	Seasonal pattern of fatalities
Traffic accident mortality of motorcyclists, pedestrians and hospital costs in São Paulo [64]	Leitão et al.	2023	Brazil	5,293 motorcyclist deaths	The average age bracket was 50-59 years old	Mostly male	NR	NR	NR	High male mortality; hospital costs significant
Temporal Instability of Motorcycle Crash Fatalities on Local Roadways [65]	Champahom et al.	2023	Thailand	2018-2020 dataset	Over 50 higher risk	Males higher risk	Helmet use decreased over time, from 51.7% in 2018 and 51.9% in 2019 to 37.5% in 2020.	The incidence of accidents involving drunk riders increased from 2019 to 2020	NR	Older age, male sex, night crashes increase risk
Factors Affecting the Number of Fatalities and Injuries in Motor Vehicle Accidents in Turkey [66]	Aşırdizer et al.	2024	Türkiye	7,199 motorcycle fatalities	Most injuries and deaths occurred between the ages of 25 and 64	Majority male (76.9%)	12,9% did not wear helmets	NR	NR	The highest risk of death was observed among individuals aged 65 and older
Profiles of Motorcycle Accident Mortality and Risk Behavior in Thai Children [67]	Chiangkhong et al.	2024	Thailand	2,775	0–14 years (64% were 10–14)	Majority male (exact % varies by cluster)	Very low (only 8% wore helmets)	Alcohol use was a contributing factor	NR	Peak fatalities occur after school hours and during school semesters; boys 10–14 most at risk, especially without helmets and under alcohol influence
On a collision course: fatal motorcycle and bicycle accidents of adolescents in Finland from 2008 to 2019 [68]	Unkuri et al.	2024	Finland	147 fatalities (77 motorcycle)	10–24 years	82% male	Low helmet use reported	NR	Head injury was the most common cause of death (62%)	Head injury was the most common cause of death; most crashes involved collision with another vehicle
The epidemiology of motorcycle-related acute traumatic brain injury: The NOMADEN study [69]	Nugroho & Masrika	2025	Indonesia	324 motorcycle TBI cases; 30 deaths	Mean 30.5 ± 16.7 years	64.8% male	8.8% helmet use	Not under alcohol influence (78.4%)	Of all RTA-related TBI patients, 91.8% (n = 324) were caused by motorcycle collision	Low GCS and delayed hospital admission predicted mortality
Motorcycle crash-related trauma in a conflict zone: Determinants of mortality and injury severity in the Middle East [70]	Gürbüz et al.	2025	Middle East	721 cases; mortality 4.2%	Most common 11–20 years	88.1% male	Not consistently reported	NR	Head and chest injuries were the primary causes of death	Mortality associated with severe head, thoracic and abdominal trauma
Trend of Injury Severity and Road Traffic-Related Mortality in an Arab Middle Eastern Country: A 12-Year Retrospective Observational Study [71]	Abulkhair et al.	2025	Qatar	12,644 RTI hospitalizations; 53 motorcycle deaths	Mean ~31.8 years	NR	NR	NR	NR	Motorcycle-related injuries increased despite overall decline in RTI mortality
Pandemic paradox: traffic mortality trends during COVID-19 in Campinas, 2019–2023 [72]	Kruger et al.	2025	Brazil	17,726 crashes; 406 deaths (including motorcyclists)	NR	Predominantly male	Low helmet compliance reported	Alcohol identified as risk factor	NR	Mortality increased despite reduced traffic; alcohol and nighttime crashes important contributors
In-Depth Analysis of Fatal Motorcycle Accidents—Case Study in Slovenia [73]	Tollazzi et al.	2025	Slovenia	138 fatal road accidents	In single-vehicle fatal crashes, riders were mainly aged 25–54 years.	92% involved male riders, 2% male passengers	NR	NR	NR	Riders causing crashes were most often aged 25–34, 45–54, and 55–64 years
Injury patterns and forensic report classification in motorcycle accidents: A large-scale retrospective study [74]	Celiksoz et al.	2026	Türkiye	2,445 cases	Mean 31.8 years	91.7% male	Helmet use low	Alcohol evaluated	The lower extremities (44.4%) and upper extremities (38.7%) were most commonly affected, followed by the head (26.0%), face (21.6%), thorax (11.2%), and abdomen-pelvis (5.4%)	Young male riders predominated; extremity and head injuries common
A Survival Analysis Based on Forensic Investigation of Motorcycle Road Traffic Accidents in the Athens Metropolitan Area During 2021–2023 [75]	Tousia et al.	2026	Greece	94 cases	Median age 40.5 years	Male (94.68%)	NR	41.43% tested positive for psychoactive substances on toxicological investigation.	77.66% of cases sustained chest injuries, 73.40% presented head injuries, 57.45%, abdominal injuries, and 22.34%, neck injuries. Extremity (upper and lower) injuries were reported in 30.85% of the cases. Multiple-site injuries were common, as they were reported in 67.02% of cases	Chest, abdominal, neck, and multiple-site injuries were associated with poorer survival in univariate analysis
Epidemiology of fatal motorcycle crashes in Ghana: a 23-year analysis of national crash data [76]	Gyaase et al.	2026	Ghana	40,322 motorcycle crashes, of which 9,213 were fatal	Fatality risk was higher in riders aged 30–39 years and especially ≥50 years.	98.91% male among fatal crash riders.	NR	NR	NR	Risk was higher in male older riders
